# An Intelligent Architecture Based on Field Programmable Gate Arrays Designed to Detect Moving Objects by Using Principal Component Analysis

**DOI:** 10.3390/s101009232

**Published:** 2010-10-15

**Authors:** Ignacio Bravo, Manuel Mazo, José L. Lázaro, Alfredo Gardel, Pedro Jiménez, Daniel Pizarro

**Affiliations:** Electronics Department, University Alcala, Escuela Politecnica, Campus Universitario, Ctra. Madrid Barcelona km. 33.6 28871, Alcala de Henares, Madrid, Spain; E-Mails: mazo@depeca.uah.es (M.M.); lazaro@depeca.uah.es (J.L.L.); alfredo@depeca.uah.es (A.G.); pjimenez@depeca.uah.es (P.J.); pizarro@depeca.uah.es (D.P.)

**Keywords:** FPGA, PCA, CMOS sensor, object detection, image processing

## Abstract

This paper presents a complete implementation of the Principal Component Analysis (PCA) algorithm in Field Programmable Gate Array (FPGA) devices applied to high rate background segmentation of images. The classical sequential execution of different parts of the PCA algorithm has been parallelized. This parallelization has led to the specific development and implementation in hardware of the different stages of PCA, such as computation of the correlation matrix, matrix diagonalization using the Jacobi method and subspace projections of images. On the application side, the paper presents a motion detection algorithm, also entirely implemented on the FPGA, and based on the developed PCA core. This consists of dynamically thresholding the differences between the input image and the one obtained by expressing the input image using the PCA linear subspace previously obtained as a background model. The proposal achieves a high ratio of processed images (up to 120 frames per second) and high quality segmentation results, with a completely embedded and reliable hardware architecture based on commercial CMOS sensors and FPGA devices.

## 1. Introduction

One of the main research areas in the field of computer vision is the automatic description of the features of a given scene [1,2]. The greater demand made by the performance of image processing algorithms, together with improved spatial resolution and the increased rate of images per second from the new CMOS sensors, means that the need for computational power is continuously increasing. If real time performance is to be achieved, the need to reduce algorithm execution time is even greater, requiring the incorporation of an operating system in the processor capable of executing deterministic tasks, which in turn increases the cost of the products and makes it more difficult to program.

It is usual for the platforms chosen to carry out these algorithms based on sequential programs, in which the only improvements currently available consist in applying multi-threading programming techniques so the power of the new multicore processors may be used. However, from a performance point of view these processing architectures are not so efficient in many applications like the digital processing of images, which normally requires a high number of operations to be handled at the bit level as quickly as possible, by processing in parallel a small number of input samples. Due to the sequential architecture of conventional computers, a notorious amount of operations cannot be performed concurrently. Another issue is the amount of data processed in each instruction, which is limited by the type and width of used communication bus and the image capture board. For this reason, when a large amount of data must be handled, the system performs slowly. This has given rise to solutions that make use of coprocessor systems that handle low level preprocessing tasks, where the amount of data to be processed is high but the operations to be carried out are simple [3]. Our proposal is to create a hardware platform for a specific purpose (designed specifically for one application), as it can produce excellent results working in an *ad-hoc* low-cost platform. In fact the FPGA used to validate the proposal could be considered as a FPGA with medium/low features (Xilinx V2P7).

The detection of both static and moving objects within a captured area is one of the more common tasks undertaken by many computer vision applications. Movement analysis is involved, among other things, in real time applications such as navigation and tracking and obtaining information about static and moving objects within a scene [4]. Movement analysis, which is closely related to the image transfer rate from the video sensor, is fundamental for addressing topics such as image sequence reconstruction, video compression, fixed image capture and multi-resolution, techniques, *etc.*

Within the field of image processing previous works have partially developed the processing algorithm of PCA using programmable devices. In [5] for example, all of the PCA is implemented on the FPGA, however the calculation of eigenvalues is implemented on a PC due to it is mathematically too complex to be implemented on the FPGA. In [6] on the other hand, a variant of PCA called a Modular PCA, applied to face recognition, has been implemented on an FPGA, as this version of PCA has a much lower volume of mathematical operations than the conventional PCA algorithm. In [7] a system based on FPGA is proposed for detecting objects known a priori by comparing their eigenvectors. However, as far as the authors know no work has been found on the detection of moving objects employing PCA that uses FPGAs as the processing element. It is important to point out that in none of the works found is PCA implemented exclusively on FPGAs, due mainly to the heavy data dependence and complex mathematical operations involve within PCA. The data dependences cause several hazards which make difficult the implementation of efficient pipeline systems. On the other hand, the mathematical operations needed by PCA algorithm, are not usual operations used for other algorithms (e.g., solving eigenproblems). Due to this fact, new specific mathematical cores have been designed for this algorithm.

These situations make difficult to segment/divide the hardware processing of the different parts of PCA. For this reason, executing PCA is normally divided between an FPGA and a PC or microprocessor [5], so that normally an *ad-hoc* HW/SW partition of the system is made, without adequately exploring the design space (HW/SW co-design methods).

One of the main contributions of this work is the FPGA implementation of the complete PCA algorithm on reconfigurable hardware; indeed it is the first work in the literature to do so. Classic sequential execution of different parts of the PCA algorithm has been parallelized. This parallelization has led to the development and implementation of seldom used alternatives for the different stages of PCA. One example is the calculation of eigenvalues and eigenvectors, matrix multiplication in hardware or calculation of a dynamic threshold for detecting moving objects. This latter issue is another major contribution of the paper because the information generated by PCA is used to detect moving objects. In this work, PCA is implemented on an FPGA to detect moving objects within a scene, based on the PCA algorithm. To achieve this, a specifically designed intelligent camera has been implemented based on a CMOS sensor and an FPGA [8]. Thanks to the design and implementation of this new proposal it can be used in any situation requiring an autonomous system (without PC).

The other sections of this paper are as follows: Section 2 sets out the mathematical foundations of the PCA algorithm applied to image processing; Section 3 describes the platform design; Section 4 presents the implementation in VHDL of the PCA algorithm on an FPGA; and finally, Sections 5 and 6 set out the results and present the conclusions respectively.

## 2. The PCA Algorithm

Principal Component Analysis (PCA) is a method that is used in different fields, such as statistics, power electronics or artificial vision. The main feature of PCA is the reduction of redundant information, retaining only information that is fundamental (principal components).

Artificial vision is a good example of a field where the PCA technique can be applied directly, as an image contains a large number of highly correlated variables (pixels). Therefore, applying the PCA technique to image processing allows us to reduce the redundant information of the initial variables and determine the degree of similarity between two or more images by analyzing only the basic features within the transformed space. This last feature is of interest as far as the detection of new objects within the scene is concerned.

### 2.1. Obtaining the principal components of an image

The PCA algorithm can be applied to images using the following steps [9,10]:

1. *Capturing M images to construct a reference model of the scene.* We identify each of *M* references image by **I***_i_* ∈ ℜ*^N×N^*, with *i =* 1*,...M* and where it is assumed that the spatial resolution of the images is N × N.
2. Each image is represented as a column vector of the dimensions *N* *^2^* × *1*
3. Calculating the mean image from the M reference images : **Ψ** ∈ ℜ^*N*^2^×1^ given for:
  (1) $$\Psi =\frac{1}{M}\cdot \sum_{i=1}^{M}I_{i}=\left[\begin{array}{c} \frac{I_{1,1}+I_{2,1}+\ldots +I_{M,1}}{M}\\ \frac{I_{1,2}+I_{2,2}+\ldots +I_{M,2}}{M}\\ \ldots \\ \frac{I_{1,N^{2}}+I_{2,N^{2}}+\ldots +I_{M,N^{2}}}{M} \end{array}\right]={\left[\begin{array}{c} \Psi_{1}\\ \Psi_{2}\\ \cdots \\ \Psi_{N^{2}} \end{array}\right]}_{N^{2}\times 1}\;\;;i=1,\ldots ,\;M$$
  where **I**_i,j_ is the *j* (*j*=1,…,*N*^2^) element of **I**_i_ image.
4. Form a matrix **A** ∈ ℜ*^N^2^×M^* (3) whose columns are the vectors **Φ***_j_* = **I***_j_* − **Ψ** (2):
  (2) $$\Phi_{i}=I_{i}-\Psi ={\left[\begin{array}{ll} I_{i,1} & -\Psi_{1}\\ I_{i,2} & -\Psi_{2}\\ .......... & ........\\ I_{i,N^{2}} & -\Psi_{N^{2}} \end{array}\right]}_{N^{2}\times 1}={\left[\begin{array}{l} \Phi_{i,1}\\ \Phi_{i,2}\\ ........\\ \Phi_{i,N^{2}} \end{array}\right]}_{N^{2}\times 1}\;\;;\;i=1,...,\;M$$
  (3) $$A=[\Phi_{1}....\Phi_{M}]={\left[\begin{array}{llll} \Phi_{1,1} & \Phi_{2,1} & .... & \Phi_{M,1}\\ \Phi_{1,2} & \Phi_{2,2} & .... & \Phi_{M,2}\\ ............ & ............ & .... & ............\\ \Phi_{1,N^{2}} & \Phi_{2,N^{2}} & .... & \Phi_{M,N^{2}} \end{array}\right]}_{N^{2}\times M}$$
5. *Obtaining the covariance matrix*, **C** ∈ ℜ*^N^2^×N^2^^* from the matrix **A** (4):
  (4) $$C=\frac{1}{M}\cdot A\cdot A^{T}$$
6. *Obtaining the associated eigenvalues and eigenvectors of the matrix* **C**. Given that matrix **A** is of the size *N^2^* × *M* and generally *N^2^* >> *M*, to reduce the number of operations that must be performed, the eigenvalues and eigenvectors of (**A***^T^* · **A)** are calculated (5):
  (5) $$A^{T}\cdot A\cdot V=\lambda I\cdot V$$
  where **V** ∈ ℜ *^M^* ^×^ *^M^* is the matrix of the eigenvectors of **A***^T^* · **A**. The eigenvalues of **C** match up with those of **A***^T^* · **A** while the eigenvectors of **C** are obtained from (6):
  (6) U=A⋅V
7. *Obtaining the principal eigenvalues.* From the eigenvalues obtained in point 6 the most significant eigenvalues *t* are selected, using for example, the criteria the normalized root mean square error (RMSE) [10,11] given by (7), that is the eigenvalues of greatest value (*λ*_1_ > *λ*_2_ > …> *λ_t_*):
  (7) $$\mathrm{RMSE}=\frac{\sum_{i=t+1}^{M}\lambda_{i}}{\sum_{i=1}^{M}\lambda_{i}}<P$$
  where *P* is the percentage of necessary eigenvalues required to achieved the most significant eigenvalues *t.*
  The transformation matrix **U***_t_* ∈ ℜ ^*N*^2^×*t*^ is given by (8) where [**u**_1_,**u**_2_,....,**u***_t_*] are the eigenvectors associated to the eigenvalues *λ_1_* > *λ_2_* > ….> *λ_t_*:
  (8) $$U_{t}=[u_{1},u_{2},\ldots ,u_{t}]$$

An important issue is the quantification of the value *M*, which is the number of captured reference images used to build the background. Theoretically, it is a good idea to employ a high *M* value that allows different lighting conditions of the same scene to be considered. However the use of a high *M* number implies a significant increase in computational load and memory storage. The features of external memory in which background images will be loaded, will determinate the size of *M*. Due to this fact, the bus width of used external memory (128 bits) and according to the results shown in [12] in our case, it has been chosen size of *M* = 8 associated to the same scene without moving obstacles and under soft natural lighting variations. Thanks to this size and CMOS features, it is possible to read from the sensor 8 pixels in each clock period.

According to the results shown in [12] in our case, a size of *M* = 8 has been chosen. Once the transformation matrix **U***_t_* has been obtained, the next step is to determine whether in a newly captured image of the scene new objects have appeared. To do this the following steps must be performed:

1. *Projection onto the transformed space*. The first step is the projection onto the transformed space using (9):
  (9) $$\Omega =U_{t}^{T}\cdot \Phi_{j}=U_{t}^{T}\cdot (I_{j}-\Psi )$$
  where **Ω** ∈ ℜ*^t^*^×1^ is characterized by a vector of dimension *t* (**Ω** = [*ω*_1_, *ω*_2_,…, *ω_t_*]*^T^*), where each component *ω_i_* represents the contribution of each eigenvector in the representation of **I**_*j*_.
2. *Recovering the projected image*. Once the image has been projected onto the transformed space using (9), then **Φ̂** ∈ ℜ^*N*^2^×1^ is recovered using (10):
  (10) $${\hat{\Phi }}_{j}=U_{t}\cdot \Omega$$
3. *Determining the existence of new objects in the scene*. Finally, the captured image is compared with the recovered image, thus obtaining what is termed the error recovery. If the result of the comparison is above a determined threshold (*Th_MD_*) it implies the presence of new objects in the scene (11):
  (11) $$\begin{array}{c} \left\|\Phi_{j}-{\hat{\Phi }}_{j}\right\|\leq {\mathrm{Th}}_{\mathrm{MD}}\to \text{there are no new objects in the scene}\\ \left\|\Phi_{j}-{\hat{\Phi }}_{j}\right\|>{\mathrm{Th}}_{\mathrm{MD}}\to \text{there are new objects in the scene} \end{array}$$
  The threshold *Th_MD_* is a dynamically obtained value that is adjusted according to the conditions of the scene.
4. *Spatial localization of the detected object*. To detect the presence of new objects it is only necessary to apply expression (11). However, if we want to know in which part of the scene the new object has appeared a localization method must be found.. With the aim of reducing the effect of noise, the value of each pixel of the captured image and that of the recovered image is averaged with that of the adjacent pixels by means of a mask of *q* × *q* elements. As a result a matrix known as an average distance map is obtained (**MD***_V_* ∈ ℜ^*N*^2^×*N^2^*^), where every one of its elements (*ε_w,i_*) corresponds to the Euclidean distance between the corresponding average pixels of the original and recovered image (12).
  (12) $$\varepsilon_{w.i}=\left\|\Phi_{w,i}-{\hat{\Phi }}_{w,i}\right\|\;\;w=1,2,\ldots N\;\;i=1,2,....,N$$

Once the **MD***_V_* has been obtained, the next step is to threshold the map so that the new objects can be found easily. To do this, a new binary image is built (**BW**) where each element is the result of a comparison between each **MD***_V_* pixel and a *Th_MD_* threshold (13):

(13) $$\begin{array}{l} {\mathrm{BW}}_{i}=255\;\;\;\text{if}\;\;\;(\varepsilon_{\mathrm{wi}})=\left\|\Phi_{\mathrm{wi}}-{\hat{\Phi }}_{\mathrm{wi}}\right\|>{\mathrm{Th}}_{\mathrm{MD}}\\ {\mathrm{BW}}_{i}=0\;\;\;\;\;\;\;\text{if}\;\;\;(\varepsilon_{\mathrm{wi}})=\left\|\Phi_{\mathrm{wi}}-{\hat{\Phi }}_{\mathrm{wi}}\right\|\leq {\mathrm{Th}}_{\mathrm{MD}} \end{array}$$

## 3. Description of the Architecture

**Figure 1.**
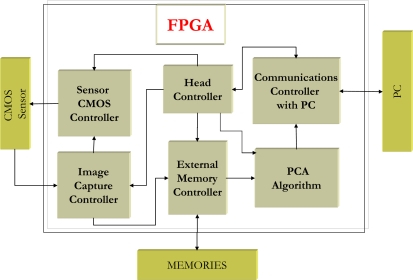
Block diagram of the internal architecture of the FPGA.

The system proposed is based on a high speed CMOS sensor (up to 500 images per second with a maximum resolution of 1,280 × 1,024 [13]) and an FPGA in which a novel design has been implemented for managing and capturing the images from the sensor, as well as executing the PCA algorithm. The system implemented on the FPGA is separated into the following logical blocks as shown in Figure 1 with green color:

- *CMOS sensor controller*: This block is responsible for implementing image demands to the CMOS sensor such as parameterizing its internal registers according to the desired configuration (images per second, exposure time, *etc.*).
- *Image capture controller*: the purpose of this block is to allow the user to select an area of interest within the image from the CMOS sensor.
- *External memory controller*: the system is equipped with a 128 MB, SDRAM memory bank that is external to the FPGA. Images from the CMOS sensor are stored in this bank.
- *Communications Controller with the PC*: this block controls the communication between the FPGA and the PC. This is used to transmit commands and results.
- *Head Controller*: This block is responsible for synchronizing the entire system so that everything works correctly and at maximum speed.
- *PCA algorithm*: This block implements the PCA algorithm and its implementation is the most important contribution of this work.

## 4. Implementing PCA on FPGAs

**Figure 2.**
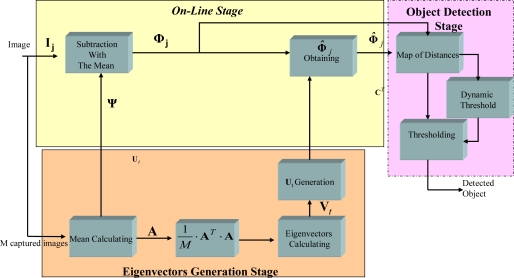
Block diagram of the PCA algorithm implemented on an FPGA.

The mathematical complexity of the operations of the PCA algorithm presented in Section 2 (calculation of eigenvectors, matrix multiplication, square roots, etc) makes it impractical to implement them directly on reconfigurable hardware. The proposal and selection of different hardware structures and computing alternatives in order to obtain an efficient solution to resolve these operations on FPGAs is essential for the PCA implementation and, thus, constitutes one of the major contributions of this paper. This section presents the hardware solution found which permits the PCA algorithm to be implemented on an FPGA. Figure 2 shows a block diagram of the PCA algorithm implemented on the FPGA, grouping the different modules into three stages: generation of eigenvectors (light yellow), on-line (light orange), and object detection (light pink). The three phases in which the PCA algorithm is divided are now described.

### 4.1. Generating the eigenvectors

The first phase of the PCA algorithm is the generation of the eigenvectors of the reduced transformation matrix **U***_t_*. This first phase includes five stages:

1. Calculating the mean of the *M* images (**Ψ** ∈ ℜ^*N*^2^×1^) and the matrix **A** ∈ ℜ^*N*^2^×*M*^ (3).
2. Obtaining the covariance matrix **C***^T^* ∈ ℜ*^M^*^×^*^M^* (5).
3. Calculating the eigenvectors of the matrix **V** ∈ ℜ*^M^*^×^*^M^* and the posterior matrix of the reduced eigenvectors **V***_t_* ∈ ℜ*^M^*^×^*^t^*.
4. Obtaining the eigenvectors of the matrix **U***_t_* ∈ ℜ^*N*^2^×*t*^ from **V***_t_* ∈ ℜ*^M^*^×^*^M^* where *t* < *M*.
5. Calculating the norms of matrix eigenvectors.

#### 4.1.1. Calculating the mean of the *M* images (**Ψ**)

**Figure 3.**
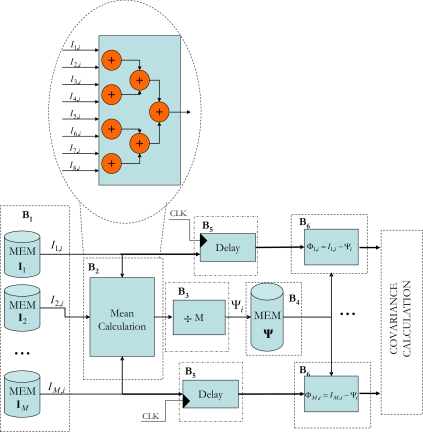
Block diagram of the proposed circuit for calculating the mean (**Ψ**) of the *M* captured images.

The hardware architecture that has been developed for this module stores the captured *M* images in an SDRAM external memory. The block shown in Figure 3 has been implemented on the FPGA, where the *M* = 8 images are stored in different memory components (B_1_). Once the eight pixels have been extracted, one for each image, the mean calculation process is initiated using a set of cascade adders (see B_2_ Figure 3). As this process takes three clock cycles and the aim is for the system to be as segmented as possible, the eight extracted pixels are inserted into a delay unit consisting of flip-flops that synchronizes the subtraction process of each pixel with that of the corresponding mean (B_6_).

#### 4.1.2. Obtaining the covariance matrix (**C***^T^*)

Generating **C***^T^* from matrix **A**, means the product of two matrices **A***^T^* · **A**, must be produced on an FPGA, which entails a complex process. In the case of the PCA the aim is to multiplex the matrix multiplication module using it to: generate the covariance matrix, generate the eigenvector matrix (**U***_t_*), project an image onto the transformed space and recover the projected image. Different approaches to the matrix multiplication have been analyzed and developed by the authors [14]. After this study, an *ad-hoc* matrix multiplier system based on a semi-systolic array proposed by the authors in [14] has been chosen because the maximum performance for PCA is achieved with this approach thanks to the possibility to reuse the system for the different types of matrix multiplication that PCA needs.

#### 4.1.3. Calculating the eigenvectors of the matrix (**V**)

The computation of eigenvalues and eigenvectors represents the greatest computational burden on the PCA algorithm. Different techniques have been proposed for obtaining the eigenvalues of a matrix using specific hardware, all of them based on recurrent methods that look to diagonalize the matrix [15,16]. Once the matrix has been diagonalized, the eigenvalues coincide with the values of the diagonal. The method proposed in [17] is the most interesting as it allows parallel processing hardware structures to be implemented [18]. For this reason, the solution developed in this work is based on the Jacobi method. A previous article by the authors [19] describes the architecture developed.

#### 4.1.4. Obtaining the eigenvectors of the matrix (**V***_t_*)

The first step in determining the most significant *t* eigenvectors is to arrange the eigenvalues and their associated eigenvectors in either ascending or descending order. This step is necessary as the Jacobi method does not generate the eigenvalues in order. To determine *t*, the largest *t* eigenvalues are found and then their associated eigenvectors are selected depending on how much bigger than the eigenvalues that have been obtained the user wants them to be (7). In this work, *bubble sort* has been used as the sorting algorithm [20].

#### 4.1.5. Obtaining the eigenvectors of the matrix **U***_t_*

To obtain the matrix **U***_t_*, according to (6), the matrix **A** must be multiplied by **V***_t_*. To do this, once again the semi-systolic *array* presented in [14] is used.

#### 4.1.6. Calculating the norms of the eigenvectors

The eigenvectors obtained in the previous stage do not possess a unit module so they must be normalized (14) **U***_tn_* according to (15):

(14) $$n_{j}=\sqrt{\sum_{i=1}^{N^{2}}{\left(u_{i,j}\right)}^{2}}\;\;\;\;\;\;j=1,\ldots ,t$$

(15) $$\begin{array}{ll} U_{\mathrm{tn}}=\frac{U_{t}}{\text{norms}}=\left(\frac{u_{1}}{n_{1}}\frac{u_{2}}{n_{2}}....\frac{u_{t}}{n_{t}}\right) & \text{norms}=[n_{1},n_{2},\ldots ,n_{t}]\in {ℜ}^{1\times t} \end{array}$$

To implement in hardware the arithmetical operations shown in expressions (14) and (15) is extremely complex as a consequence of the square root, and it also uses a large amount of resources. To avoid calculating the square root when calculating **Φ̂***_j_* it is only necessary to express this matrix in accordance with the squared norm, as shown in (16):

(16) $${\hat{\Phi }}_{j}=U_{\mathrm{tn}}\cdot \Omega =U_{\mathrm{tn}}\cdot U_{\mathrm{tn}}^{T}\cdot \Phi_{j}=\frac{U_{t}\cdot U_{t}^{T}\cdot \Phi_{j}}{{\text{norms}}^{2}}$$

### 4.2. The on-line stage

If there is a new object in the captured image, with respect to the reference scene, it is determined during the *on-line* stage. For this to be done, the new captured image is projected onto the transformed space so that it can be recovered later and studied to determine whether or not there is a new object in the scene. To do this the following steps are followed:

1. *Subtraction of the mean of the present image*: If **I***_j_* is the captured image, **Φ***_j_* is obtained.
2. *Projecting* **Φ***_j_* *onto the transformed space and obtaining* **Φ̂***_j_*: With the aim of reaching the maximum concurrence possible when executing (16), first the $U_{t}^{T}\cdot \Phi_{j}$ product is performed and this result is divided by the squared norms ( $\frac{\left(U_{t}^{T}\cdot \Phi_{j}\right)}{{\text{norms}}^{2}}$), and finally $U_{t}\cdot \left(\frac{\left(U_{t}^{T}\cdot \Phi_{j}\right)}{{\text{norms}}^{2}}\right)$ product is performed.
3. *Determining the recovery error*: In this final stage, the degree of similarity between **Φ***_j_* and **Φ̂***_j_*is evaluated.

**Figure 4.**
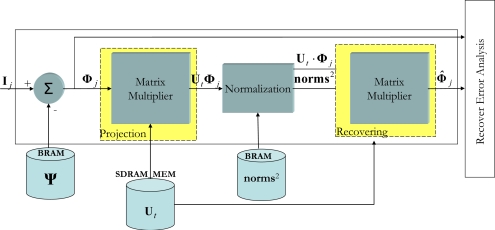
Block diagram of the modules of the design in VHDL of the on-line stage of the PCA.

Figure 4 shows the VHDL encoded modular design of this *on-line* stage. With respect to the internal workings of the system shown in Figure 4, this starts when a new vector image **I***_j_* is captured and later stored in the external memory so that the system has an initial latency of one image. As explained earlier, once **Φ***_j_* has been obtained the next step is to produce the $U_{t}^{T}\cdot \Phi_{j}$. To do this, the semi-systolic array for matrix multiplication is used [14]. It is important to point out at this point that the execution time of the Matrix Multiplier depends on the number of significant eigenvalues (*t*). In accordance with the percentage of significant eigenvalues (see (7)), a value of *t* equals 6 has been decided upon. This reduction introduces a recovery error (*ε*) (17), after analyzing 1,000 images it could be seen that the induced error is approximately 1%:

(17) $$\varepsilon =\left\|\Phi_{j}-{\hat{\Phi }}_{j}\right\|=\sqrt{\sum_{i=1}^{N^{2}}{\left(\Phi_{j_{i}}-{\hat{\Phi }}_{j_{i}}\right)}^{2}}$$

Once the first results from the $U_{t}^{T}\cdot \Phi_{j}$ product have been obtained, the next step is to divide these results by **norms^2^**. As each component of $U_{t}^{T}\cdot \Phi_{j}$ is generated in one clock cycle, given that they are output by the semi-systolic array, they are divided by the corresponding squared norm.

To perform the division operation on an FPGA, there are basically two possibilities: either design a division unit specifically for that purpose, or use a coordinate rotation digital computer (CORDIC) algorithm [21]. In this work the latter option has been chosen, as it consumes fewer resources than the former. Dividing two numbers is feasible in CORDIC if it is used in vectorization mode with a linear coordinate system [22]. To do so, a division module based on a parallel CORDIC architecture has been implemented.

When the first component of the division has been obtained, the next step to be performed in (16) is to obtain **Φ̂***_j_*. Once again, to perform this fourth matrix multiplication, the semi-systolic array described in [14] is used.

### 4.3. Detecting new objects in the scene

**Figure 5.**
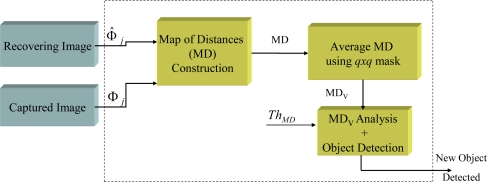
Proposal for the system consisting of the construction of the MD, detection of new objects and the updating of the background model.

This section presents the solution developed for implementing an identification of new objects from the error recovery (*ε*) system in reconfigurable hardware (17). It proposes the building of an error recovery map or Map of Distances (**MD**) that will permit the new objects to be located spatially. The size of this map of distances will coincide with the size of the image, where each of its positions is the pixel to pixel Euclidean distance between **Φ̂***_j_* and **Φ***_j_*. A new Map of Distances (**MD***_V_*) will be built in order to reduce the noise effect. The final detection of moving obstacles will be obtained using the dynamic threshold *Th_MD_* (11). Calculating *Th_MD_* presents difficulties as it must be adaptable and its value depends on both the features of the scene under analysis and the lighting conditions. For this reason, in this section we present a new method for dynamically calculating the threshold that minimizes the false detection of new objects within the scene of interest. Figure 5 shows a block diagram of this proposal for detecting objects from **Φ̂***_j_* and **Φ***_j_* (green blocks). Next the hardware solution implemented in each block of Figure 5 is presented.

#### 4.3.1. Constructing the Map of Distances (MD) and the Map of average Distances (MD_V_)

The Map of Distances **MD** is obtained from (18), *ε*′*_j_* being the square of the Euclidean distance between each component Φ*_ji_* ∈ **Φ***_j_* and Φ̂*_ji_* ∈ **Φ̂***_j_ i* = 1,....*N*^2^ (for images of the size *N* × *N*):

(18) $$\varepsilon_{i}^{\prime }={\left\|\Phi_{j_{i}}-{\hat{\Phi }}_{j_{i}}\right\|}^{2}\;\;\;i=1,\ldots N^{2}$$

Working with the square of the Euclidean distance rather than the Euclidean distance (17), facilitates the design of hardware associated with this function, as it avoids the need to perform the square root operation. As such, to obtain **MD** only requires one subtraction and one multiplication operation, so that with an adder/subtraction block and a multiplier connected in cascade the segmented execution of (18) can be performed.

Once the initial components of **MD** have been generated, the generating of the map of average distances (**MD***_V_*) can be started. The use of a mask of *q* × *q* components is proposed that averages the pixels adjacent to **MD**, applying a 2D low-pass filter. The components that make up the map **MD***_V_* are *ε*′*_v_i,w__*; *i*, *w* = 1,....,*N*.

To provide a compromise value to the size of mask *q*, different sizes applied to different maps **MD** have been simulated; all of them are fixed point encoded. The size chosen for *q* is 3, given that it provides the algorithm with a certain degree of robustness and reliability, and few hardware resources are required.

To implement the averaging function with masks of *q* × *q* (3 × 3) on the adjacent pixels the corresponding convolution function is implemented [23]. To select the best alternative for hardware implementation, several proposals for convolutions have been designed [23], evaluating at all times the execution time as well as how much of the FPGA’s internal resources are consumed.

To perform the convolution between a matrix and a generic mask, nine multiplication operations and eight accumulation operations must be performed for each resulting component. However, when all the coefficients of the mask have been identified, as happens in our case, another way of performing the convolution is according to (19), whereby one that reduces the number of multiplications to one. In this way, to obtain each *ε*′*_v_i,w__* component of the **MD***_V_* it is necessary to perform a nine component sum backlog and one multiplication for the equivalent factor in fixed point:

(19) $$\varepsilon \prime_{\nu_{i,w}}=1/9\cdot \left(\varepsilon \prime_{i-1,w-1}+\varepsilon \prime_{i-1,w}+\varepsilon \prime_{i-1,w+1}+\varepsilon \prime_{i,w-1}+\varepsilon \prime_{i,w}+\varepsilon \prime_{i,w+1}+\varepsilon \prime_{i+1,w-1}+\varepsilon \prime_{i+1,w}+\varepsilon \prime_{i+1,w+1}\right)$$

#### 4.3.2. Detecting objects from the **MD***_V_* map

**Figure 6.**
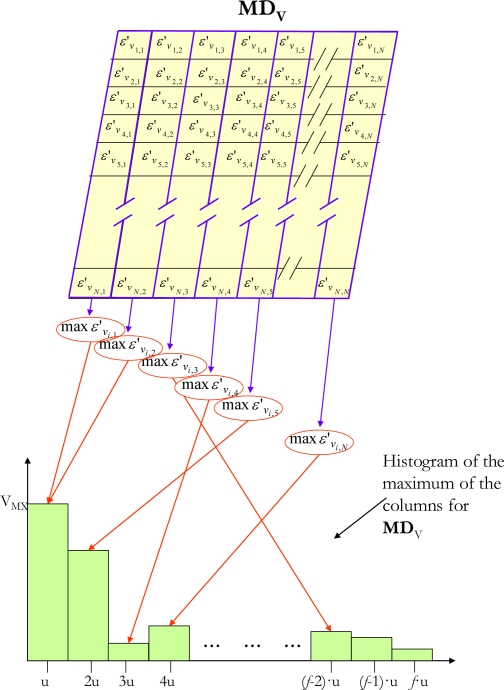
Example of histogram construction of the maximum of the columns for an average map of distances (**MD***_V_*).

Once the map of average distances (**MD***_V_*) has been obtained, the next step is to analyze the map to evaluate whether or not there are new objects in the scene of interest. To do so, a threshold *Th_MD_* is obtained, which, when applied to **MD***_V_* makes it possible to perform the segmentation and as a consequence detect the presence of new objects. The value of *Th_MD_* must be dynamic as its value must adapt, amongst other factors, to changes in light within the scene. In order to obtain this dynamic *Th_MD_* different alternatives have been proposed, [12,24,25]. Our proposal calculates the histogram (with *f* intervals) of the maximum Euclidean distances of each column of the **MD***_V_* (Figure 6) and then obtains the dynamic threshold *Th_MD_* from the histogram. This algorithm, implemented on an FPGA, generates excellent results, as will be seen later in the results section.

Analyzing the information supplied by the histogram on the maximums of the **MD***_V_* columns, it can be seen how most of the maximum Euclidean distances represented are concentrated in the lower intervals. However, when a new object appears in the scene being studied, the maximum Euclidean distances of the **MD***_V_* columns where the object is located are expressed by a valley in the histogram. If there is no new object in the scene, then the valley does not appear. On the basis of this last feature of the histogram, to threshold **MD***_V_* it is necessary to find the value of *Th_MD_* that makes it possible to discriminate between the new object and the background. The minimum value of *Th_MD_* needed to correctly detect new objects must be the same as the value of the histogram interval that contains the valley associated with the new object.

**Figure 7.**
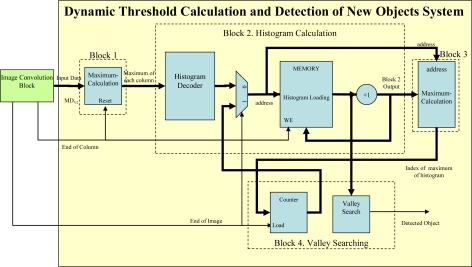
Block diagram on an FPGA of the dynamic threshold calculating system for detecting new objects.

The hardware to perform the threshold is shown in Figure 7. Each block in Figure 7 is described below:

- *Block 1*: this block is responsible for calculating the maximum of each column of the map of distances **MD***_V_*. Internally it consists of a single register that stores the maximum value and a comparator that evaluates whether the new data is bigger or smaller than the stored temporal maximum.
- *Block 2*: After calculating the maximums of the columns of the **MD***_V_*, *Block 2* is responsible for building the histogram of the maximums of the columns. It is executed in parallel with *Block 1* once the maximum of the first column has been obtained. Every time a maximum is obtained the histogram interval that belongs to that maximum must be looked for and its accumulator increased by 1.
- *Block 3*: This module, which is executed when *Block 2* generates the first data, is responsible for calculating the maximum values of the histogram (V_MX_ of Figure 6). This block works as follows: every time the maximum of a column is obtained in *Block 2*, a new value is added to the corresponding histogram interval and the number of the histogram interval with the maximum accumulated value is updated. At the same time, in *Block 3* the increased value is evaluated to see whether it is the largest. If it proves to be so, then it is stored so that it can be compared with the following output from *Block 2* and its memory address, which gives the location of the new maximum, generated by *Block 2* is also stored.
- *Block 4*: Finally, this component is responsible for looking for valleys in the histogram once Block 2 and Block 3 have finished.

To find a valley, a hardware block has been designed to check the memory of *Block 2*, which contains the histogram of the maximums of the columns of **MD***_V_*. The counter starts from the address stored in *Block 3*, that is to say, the address of the histogram interval with the maximum accumulated value. To find a valley, it is only necessary to find a value in the memory that is bigger than the one stored in the position before it. If no local minimum exists the system will increase the threshold (checking the intervals defined by the histogram) until it considers that the threshold is situated in the extreme interval and then classifies all the pixels in the image as belonging to the background. The number of histogram intervals (*f*) has been empirically set at 10, as with this value the developed proposal works correctly.

## 5. Results

This section sets out the results obtained in detecting new objects with a FPGA running PCA algorithm. All the images presented in this work have been captured by an “intelligent camera” described in [8].

From a quantitative point of view, in calculating the execution time of the entire proposal presented in this work (T_PCA_TOTAL_) from the moment the first *M* images are captured, the total time consumed is given by (20), with Table 1 giving a description of each of the times in (20):

(20) $$T_{\mathrm{PCA}\_\mathrm{TOTAL}}=T_{\mathrm{GEN}\_\mathrm{WR}\_U}+T_{\mathrm{IMAGE}}+L_{\mathrm{MEM}}+T_{\mathrm{OBJ}}$$

**Figure 8.**
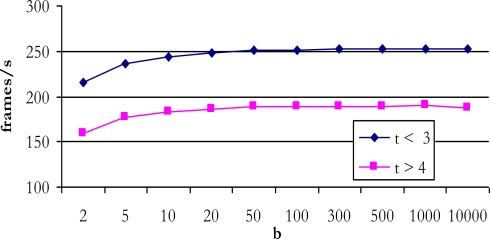
Ratio of images achieved per second with *b* ≠ 1.

When it comes to calculating the number of complete clock cycles employed by T_PCA_TOTAL_, the value obtained is not constant as it depends on the number of significant eigenvectors, the size of the matrix and the number of Jacobi algorithm iterations, as explained in [19]. Adjusting the expression (20) for six eigenvectors (worst case), capturing eight images (256 × 256 pixels) to build a reference model (*M* = 8), an internal data width of 18 bits (*n* = 8) and 23 iterations for the Jacobi algorithm the value obtained in clock cycles is:

(21) $$T_{\mathrm{PCA}\_\mathrm{TOTAL}}=131076\;\;T_{\mathrm{CLK}\_\mathrm{CAMERA}}+526939\;\;T_{\mathrm{CLK}}$$

where T_CLK_CAMERA_ is the signal period of the CMOS sensor’s clock and T_CLK_ the FPGA’s master clock. Clock Camera is generated by the FPGA using a DCM (digital clock management) block. Thanks to this element and a bank register managed for a FSM (finite state machine), both clocks working rightly. To obtain a ratio of the number of images the system processes, if the CMOS sensor’s clock (T_CLK_CAMERA_) is 66 MHz and the FPGA’s master clock is 100 MHz (frequency reached once the entire system has been implemented) a minimum of 121 images of 256 × 256 pixels have been processed per second. This ratio increases notably if any of the following situations occur:

- *Number of significant eigenvectors (t) under four*. In this case the number of matrix multiplication operations (6), (9) and (10) are notably reduced. In this way the new T_PCA_TOTAL_ value would reach an equivalent image per second ratio of 189.
- *Selective actualization*. Cadence is another very important factor that conditions the number of images processed per second when updating the eigenvectors of the matrix (background model). If the eigenvalues of the matrix are not continuously updated, but between one update and another *b* images pass, the new ratio of images per second obtained is shown in Figure 8.
- As may be seen from this figure, from *b* = 100 onwards, independent of the number of significant eigenvectors, the system reaches its maximum value at around 250 images per second for *t* ≤ 3 and around 190 for *t* ≥ 4. This is because the system segmentation is at its most efficient at this number of images. In Table 2 a summary of the final amount of resources consumed by the different blocks implemented on the Xilinx FPGA is presented. It is important to point out that due to the limited resources of the FPGA every attempt has been made to optimise the design at all times, with the aim of reducing the use of internal resources. Thanks to this, from a number of BRAM (block RAM) components and *slices* point of view, it has been possible to implement the entire system on a medium to low range FPGA like the Xilinx XC2VP7.

With respect to the frequency of the FPGA clock, according to the reports generated by the implementation tool, a maximum value of 1,124 MHz for the entire FPGA is assured. However, the master frequency chosen for our design is 100 MHz as from this value all the other necessary frequencies can be generated (the camera and external memory frequencies).

**Figure 9.**
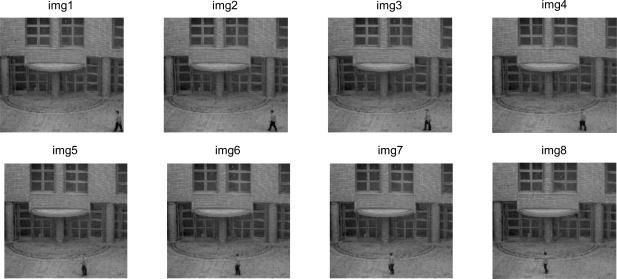
Sequence of images captured to determine new objects.

As for the real results obtained, Figure 9 shows images captured with the developed platform [8] with an initial resolution of 1,280 × 1,023 reducing their size to 256 × 256 by applying a *binning* process on the FPGA. This sequence was captured in the grounds of the University of Alcala where the distance between the objects to be detected, in this case people and the camera, is 25 meters.

**Figure 10.**
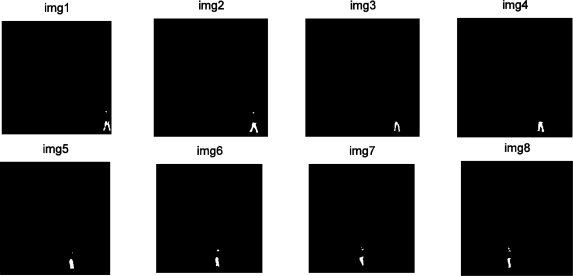
Sequence of images detected to determine a new object from those captured in Figure 9.

Figure 10 shows the detection that was performed. The proposed design has been tested with a bank of 1,000 images captured under moderate lighting conditions in outside environments. The accuracy achieved in the test was remarkable (around 97% of true matches). Despite the promising results for an embedded architecture, it is widely known that when using PCA for modelling strong illumination changes in the intensity values of the image require a high amount of PCA vectors to train the background. Besides, due to the fact that illumination changes are non-linear variations of the intensity, the PCA subspace cannot model such variations properly, which could increase the number of false detections. In a near future the proposal can be easily applied to other colour spaces, such as the light invariant space proposed in [26], which maps a RGB image to a scalar image where same surfaces under different illuminations are mapped to the same intensity value.

## 6. Conclusions

This work presents a new image capture and processing system implemented on FPGAs for detecting new objects in a scene, starting from a reference model of the scene. To achieve this, the Principle Component Analysis (PCA) technique has been used. The main objective is to parallelize it in order to achieve a concurrent execution which will enable processing speeds of around 120 images per second to be reached. This processing speed, including all stages included in the PCA technique (calculating eigenvalues and eigenvectors, projection and recovery of images to/from the transformed space, obtaining map of distances, *etc.*) responds to the requirements of many applications, where the goal is the detection of new objects in the scene, even in those cases where, for a variety of reasons, (changes in lighting for example) a continuous update of the background model is required. The proposed solution is a significant improvement on other hybrid solutions based on the use of a PC and an FPGA [5]. The complete integrated development of the PCA algorithm on an FPGA was a task that until now had not been achieved or performed, at least according to our thorough review of related work done on this topic. Thanks to the designed solution new applications with PCA algorithm could be implemented for new proposals or applications.

## Figures and Tables

**Table 1.** Description of the partial times of T_PCA_TOTAL_.

T_GEN_WR_U_	Time the FPGA takes to generate and write in SDRAM the eigenvectors of the matrix **U***_t_*.
T_IMAGE_	Time employed in capturing a new image and its subsequent writing in SDRAM.
L_MEM_	Latency of the SDRAM memory, from the time it gives the order to read an image until the first data is received.
T_OBJ_	Time consumed in detecting new objects after the recovered image (**Φ̂***_j_*) has been obtained from the transformed space

**Table 2.** Summary of all the resources consumed by the entire developed system on a XC2VP7.

**Area (Slices)**	**BRAM**	**Multipliers**	**f_CLKMAX_**
4225 (86%)	40 (91%)	43 (98%)	112,4MHz
